# Data on SVCT2 transporter expression and localization in cancer cell lines and tissues

**DOI:** 10.1016/j.dib.2019.103972

**Published:** 2019-05-06

**Authors:** Francisco J. Roa, Eduardo Peña, Eveling Inostroza, Kirsty Sotomayor, Mauricio González, Francisco A. Gutierrez-Castro, Michelle Maurin, Karen Sweet, Claire Labrousse, Marcell Gatica, Carlos F. Aylwin, Pamela Mendoza, Mafalda Maldonado, Carolina Delgado, Jaime Madariaga, Jessica Panes, Tiare Silva-Grecchi, Ilona I. Concha, Gustavo Moraga-Cid, Alejandro M. Reyes, Carola Muñoz-Montesino, Juan Carlos Vera, Coralia I. Rivas

**Affiliations:** a Departamento de Fisiopatología, Facultad de Ciencias Biológicas, Universidad de Concepción, Barrio Universitario s/n, PO Box 160C, Concepción, Chile; b Departamento de Especialidades Médicas, Facultad de Medicina, Universidad de Concepción, Barrio Universitario s/n, PO Box 160C, Concepción, Chile; c Departamento de Fisiología, Facultad de Ciencias Biológicas, Universidad de Concepción, Barrio Universitario s/n, PO Box 160C, Concepción, Chile; d Instituto de Bioquímica y Microbiología, Facultad de Ciencias, Universidad Austral de Chile, Campus Isla Teja, PO Box 567, Valdivia, Chile

## Abstract

The data presented in this article are related to the research paper entitled “Increased expression of mitochondrial sodium-coupled ascorbic acid transporter-2 (mitSVCT2) as a central feature in breast cancer”, available in Free Radical Biology and Medicine Journal [1]. In this article, we examined the SVCT2 transporter expression in various breast cancer cell lines using RT-PCR and Western blot assays. In addition, we analyzed the subcellular localization of SVCT2 by immunofluorescence colocalization assays and cellular fractionation experiments. Finally, an analysis of different cancer tissue microarrays immunostained for SVCT2 and imaged by The Human Protein Atlas (https://www.proteinatlas.org) is presented.

**Table:** Specifications Table *[please fill in right-hand column of the table below]*

Subject area	*Biology*
More specific subject area	*Cancer biology*
Type of data	*Figures and Images*
How data was acquired	*RT-PCR, Western blot, Microscopy and Atlas analysis.*
Data format	*Analyzed.*
Experimental factors	*Breast cancer cell lines were cultured in standard conditions.*
Experimental features	*RT-PCR and western blot were performed to analyze SVCT2 expression.* *Immunofluorescence and cellular fractionation followed by western blot were performed to analyze SVCT2 subcellular localization.* *Several cancer tissue microarrays immunostained for SVCT2 and imaged by The Human Protein Atlas were analyzed.*
Data source location	*Concepción, Chile.*
Data accessibility	*Data is provided in this article.*

**Table:** 

**Value of the data** • This research analyzes the expression and subcellular localization of SVCT2 transporter in human cancer cells. • Data from this work describe the mitochondrial localization of SVCT2 transporter in breast cancer cell lines. • Analysis of numerous tissue microarrays contained in The Human Protein Atlas reveal the intracellular expression of SVCT2 in different cancer tissues. • These data are relevant in cancer biology research, especially for the understanding of vitamin C mitochondrial role and its use in therapeutic procedures for human cancer.

## 1 Data

**Fig. 1:**
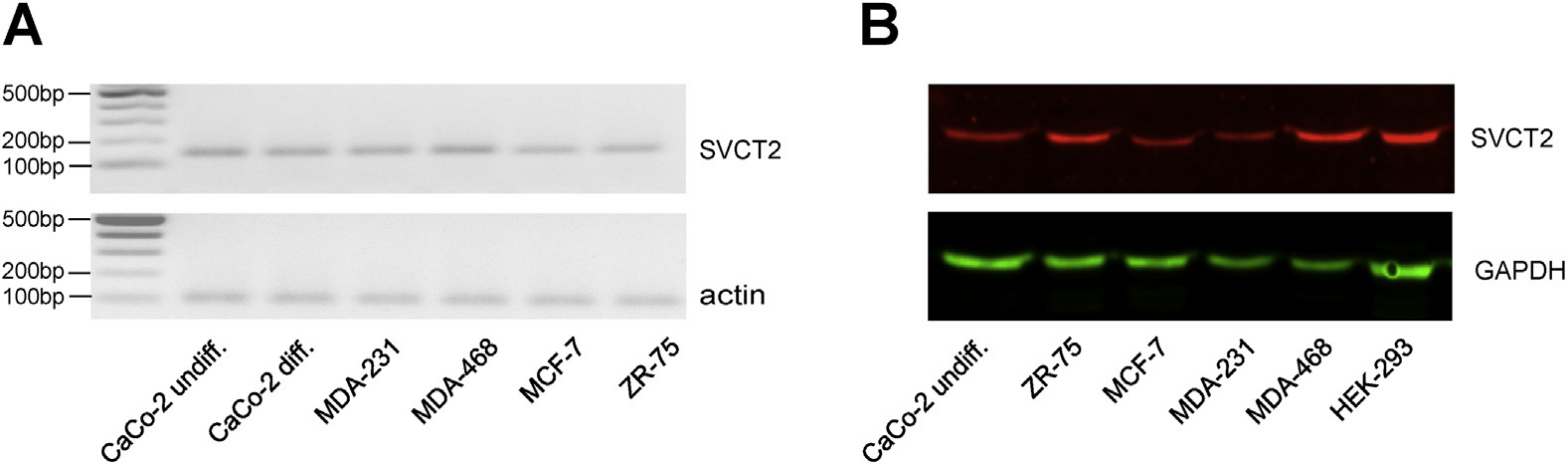
SVCT2 expression in breast cancer cell lines. (A) RT-PCR for SVCT2 expression in breast cancer cells. cDNA was synthesized from total RNA isolated from four breast cancer lines and control cells (undifferentiated and differentiated CaCo-2 cells), followed by amplification by PCR, separation by agarose gel electrophoresis and visualization by ethidium bromide staining. Actin was used as an internal control. (B) Western blot for SVCT2 protein expression in breast cancer cells. Total extracts were prepared from four breast cancer lines and control cells (undifferentiated CaCo-2 and HEK-293 cells) and immunoblotted with anti-SVCT2 antibody. GAPDH was used as a loading control.

**Fig. 2:**
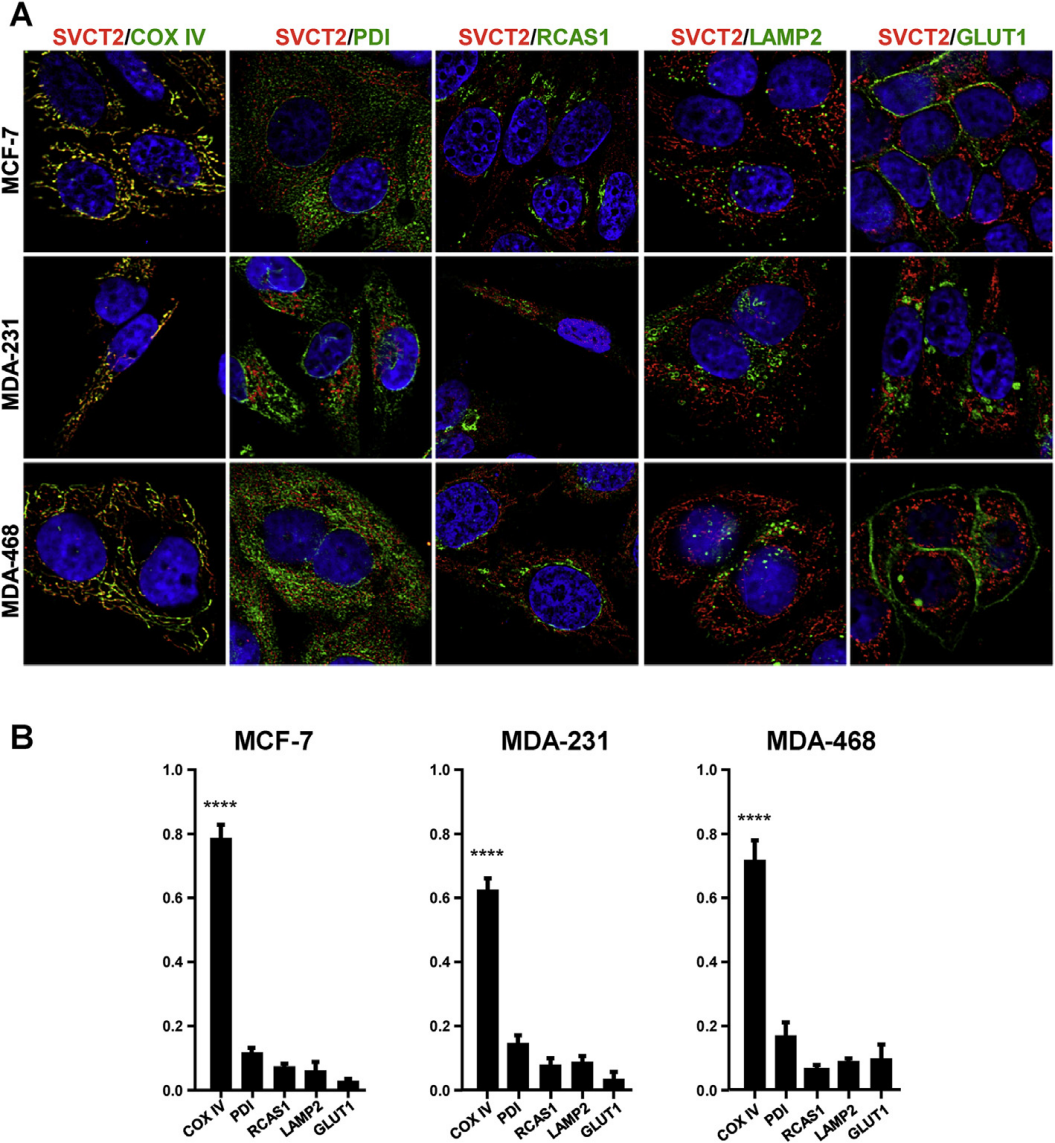
Colocalization of SVCT2 with organelle markers in breast cancer cell lines. (A) Immunofluorescence was performed in MCF-7, MDA-231 and MDA-468 cell lines. Cells were stained with anti-SVCT2 simultaneous with anti-COXIV, anti-LAMP2, anti-PDI, anti-RCAS1 or anti-GLUT1; and stained with Cy3 and FITC secondary antibodies. Samples were observed by confocal microscopy and 0.2-μm successive images were obtained along the z axis for further deconvolution analysis. (B) Pearson colocalization analysis in ImageJ using the JaCop plugin.

**Fig. 3:**
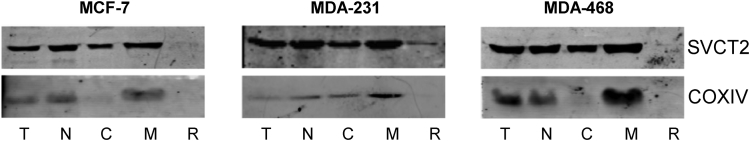
SVCT2 expression in mitochondrial fraction of breast cancer cell lines. MCF-7, MDA-231 and MDA-468 cell lines were homogenized and fractionated by differential centrifugation, and the different fractions (T: total homogenate; N: nuclear fraction; C: cytoplasmic fraction; M: mitochondria; R: endoplasmic reticulum) were immunoblotted with anti-SVCT2 and anti-COXIV antibodies.

**Fig. 4:**
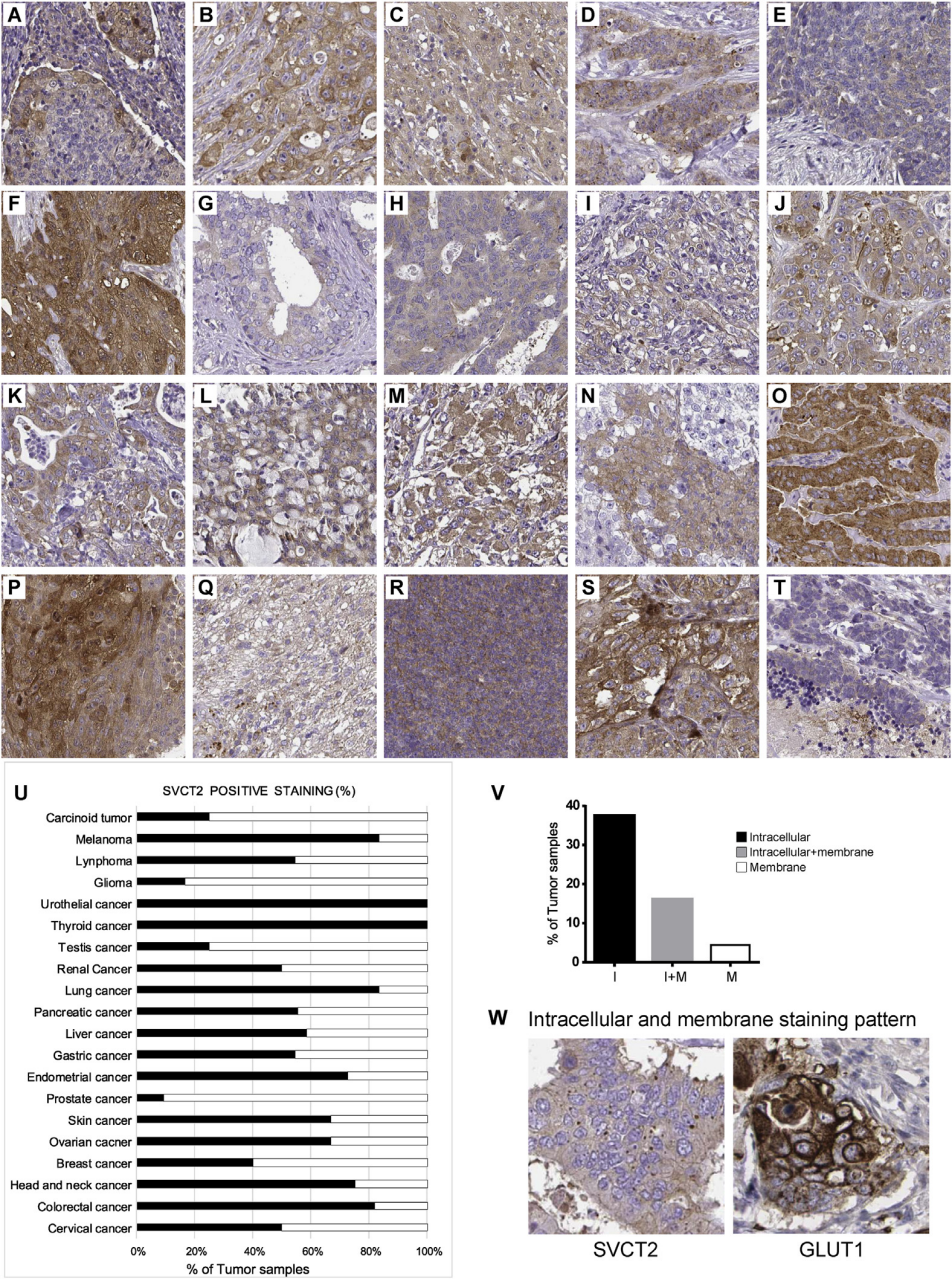
SVCT2 expression in different cancer tissues. Various tissue microarrays processed, immunostained for SVCT2 transporter and imaged by The Human Protein Atlas in different cancer tissues: cervical (A), colorectal (B), head and neck (C), breast (D), ovarian (E), skin (F), prostate (G), endometrial (H), stomach (I), liver (J), pancreatic (K), lung (L), renal (M), testis (N), thyroid (O), urothelial (P), glioma (Q), lymphoma (R), melanoma (S) and carcinoid (T). (U) Percentage of positive SVCT2 samples among different cancer types. (V) Percentage of SVCT2 positive samples with intracellular (I), intracellular plus membrane (I+M) and membrane (M) SVCT2 staining patterns. (W) Staining pattern comparison between membrane staining of GLUT1 vs. intracellular staining of SVCT2. (Images adapted from the protein atlas website https://www.proteinatlas.org/ENSG00000089057-SLC23A2/pathology).

Here we report experimental data on SVCT2 transporter expression and localization in human cancer cell lines and tissues. Analysis of SVCT2 expression by RT-PCR and Western blot in four breast cancer cell lines are shown (Fig. 1). Immunofluorescence colocalization assays for SVCT2 with various organelle markers in the breast cancer cells MCF-7, MDA-231 and MDA-468 revealed mitochondrial localization of SVCT2 (Fig. 2). This was confirmed with cellular fractionation experiments followed by Western blot for various subcellular fractions (Fig. 3). We finally analyzed the SVCT2 expression in the Human Protein Atlas, a public database containing a tissue-based map of the human proteome. Examination of numerous tumor samples of different origins (Fig. 4A-U) revealed a mainly intracellular SVCT2 immunoreactivity (Fig. 4V), in contrast with similar samples stained with anti-GLUT1 indicating a plasma membrane staining pattern (Fig. 4W).

## 2 Experimental design, materials and methods

### 2.1 Cell culture

Breast adenocarcinoma cell lines ZR-75, MCF-7, MDA-231 and MDA-468 were grown in DMEM:F12 (1:1) medium. Colon carcinoma cell line CaCo-2 was grown in DMEM medium. Embryonic kidney cell line HEK-293 was grown in DMEM-high glucose medium. All media were supplemented with 10% fetal bovine serum, 1% nonessential amino acids, 1% l-glutamine, penicillin/streptomycin (100 U/ml) and fungizone.

### 2.2 RT-PCR analysis

PCR experiments were performed using the protocol described in Ref. [2]. Briefly, total RNA was extracted from different cell lines using an RNeasy mini kit (Qiagen). cDNA was synthesized with the AffinityScript Multi-Temp RT (Agilent) plus oligo(dT) and random primers. PCR reaction mixture included specific primers for SVCT2 (or Actin) amplification, Taq 2 Master Mix (New England BioLabs) and 100 ng of cDNA. Amplification products were examined by electrophoresis on 1.5% agarose gels and visualized by ethidium bromide staining.

### 2.3 Western blot analysis

Immunoblotting experiments were performed using the protocol described in Ref. [1]. Briefly, total extracts were prepared from different cell lines, quantified, separated by SDS-PAGE and transferred to PVDF membranes. Detection was performed using anti-SVCT2 and anti-GAPDH primary antibodies (Santa Cruz Biotechnology, Inc) followed by Alexa 680 and Alexa 790 secondary antibodies (Jackson Immunoresearch). Membranes were scanned and analyzed with an Odyssey CLx Imaging system (LI-COR Biosiences).

### 2.4 Immunolocalization

Immunofluorescence experiments were performed using the protocol described in Ref. [1]. Briefly, cells were fixed with paraformaldehyde, permeabilized with Triton X-100, blocked with bovine serum albumin, incubated with primary antibodies and incubated with secondary antibodies, before observation by confocal microscopy. The following primary antibodies were used: anti-SVCT2 and anti-RCAS1 (golgi protein) from Santa Cruz Biotechnology, Inc.; anti-COXIV (inner mitochondrial membrane), anti-LAMP2 (lysosome), anti-PDI (endoplasmic reticulum membrane) and anti-GLUT1 from Abcam, Inc. Three different secondary antibodies were used: fluorescein-labeled anti-mouse IgG and Cy3-labeled anti goat-IgG from Jackson Immunoresearch; fluorescein-labeled anti-rabbit IgG from Dako. Samples were observed using an Olympus IX81 fluorescence microscope with a DSU (Disk Scanning Unit) spinning disk confocal system and images were obtained by a HAMAMATSU ORCA-R2 camera controlled by Olympus Xcellence R software. After deconvolution, images were subjected to Pearson colocalization analysis. Statistical correlation of the colocalization index among the different organelle markers was established by one-way Anova, followed by Tukey test for multiple comparison.

### 2.5 Mitochondrial isolation

Cellular fractionation and mitochondria purification were performed using the protocol described in Ref. [1]. Briefly, mitochondria were isolated from different cell lines by differential centrifugation with all steps carried out at 4 °C [3]. The final pellet containing mitochondria and different fractions obtained (T: total homogenate; N: nuclear fraction; C: cytoplasmic fraction; R: endoplasmic reticulum) were separated by SDS-PAGE, transferred to PVDF membranes and immunodetected with anti-SVCT2 and anti-COXIV antibodies.
